# Effectiveness of eHealth interventions for reducing mental health conditions in employees: A systematic review and meta-analysis

**DOI:** 10.1371/journal.pone.0189904

**Published:** 2017-12-21

**Authors:** Elizabeth Stratton, Amit Lampit, Isabella Choi, Rafael A. Calvo, Samuel B. Harvey, Nicholas Glozier

**Affiliations:** 1 Brain and Mind Centre, Sydney Medical School, University of Sydney, Sydney, Australia; 2 School of Psychiatry, University of Sydney, Sydney, Australia; 3 School of Electrical and Information Engineering, University of Sydney, Sydney, Australia; 4 School of Psychiatry, University of New South Wales, Sydney, Australia; 5 Black Dog Institute, Sydney, Australia; 6 St George Hospital, Sydney, Australia; Swansea University, UNITED KINGDOM

## Abstract

**Background:**

Many organisations promote eHealth applications as a feasible, low-cost method of addressing mental ill-health and stress amongst their employees. However, there are good reasons why the efficacy identified in clinical or other samples may not generalize to employees, and many Apps are being developed specifically for this group. The aim of this paper is to conduct the first comprehensive systematic review and meta-analysis evaluating the evidence for the effectiveness and examine the relative efficacy of different types of eHealth interventions for employees.

**Methods:**

Systematic searches were conducted for relevant articles published from 1975 until November 17, 2016, of trials of eHealth mental health interventions (App or web-based) focused on the mental health of employees. The quality and bias of all identified studies was assessed. We extracted means and standard deviations from published reports, comparing the difference in effect sizes (Hedge’s g) in standardized mental health outcomes. We meta-analysed these using a random effects model, stratified by length of follow up, intervention type, and whether the intervention was universal (unselected) or targeted to selected groups e.g. “stressed”.

**Results:**

23 controlled trials of eHealth interventions were identified which overall suggested a small positive effect at both post intervention (g = 0.24, 95% CI 0.13 to 0.35) and follow up (g = 0.23, 95% CI 0.03 to 0.42). There were differential short term effects seen between the intervention types whereby Mindfulness based interventions (g = 0.60, 95% CI 0.34 to 0.85, n = 6) showed larger effects than the Cognitive Behaviour Therapy (CBT) based (g = 0.15, 95% CI 0.02 to 0.29, n = 11) and Stress Management based (g = 0.17, 95%CI -0.01 to 0.34, n = 6) interventions. The Stress Management interventions however differed by whether delivered to universal or targeted groups with a moderately large effect size at both post-intervention (g = 0.64, 95% CI 0.54 to 0.85) and follow-up (g = 0.69, 95% CI 0.06 to 1.33) in targeted groups, but no effect in unselected groups.

**Interpretation:**

There is reasonable evidence that eHealth interventions delivered to employees may reduce mental health and stress symptoms post intervention and still have a benefit, although reduced at follow-up. Despite the enthusiasm in the corporate world for such approaches, employers and other organisations should be aware not all such interventions are equal, many lack evidence, and achieving the best outcomes depends upon providing the right type of intervention to the correct population.

## Introduction

Mental health problems in the workforce are common and have a substantial impact on employee wellbeing, productivity, absenteeism, compensation claims, and the social welfare systems [1–3]. Reported annual productivity losses to organisations and workforces caused by mental health conditions are estimated at $225US billion in the USA [4] and £13 billion in the UK [5].

The majority of mental ill-health seen in the workforce is due to common mental disorders, most notably, depression and anxiety [6–8], accounting for up to 12% of the Australian working population [9]. However, common mental disorders may go unnoticed in the workplace, as they can often be characterized as work stress or other stress related conditions [7]. More severe mental illness is less common in the workplace, due to lower levels of unemployment in those with severe mental illness [10].

Over the recent decades, organisations have increasingly recognised the importance of maximizing employee health, both for ethical reasons, to improve productivity, meet legislation changes and reduce their cost burden [11,12]. While physical health promotion interventions are well established in many workplaces [13], workplace mental health interventions have lagged. Despite mental illness now being the leading cause of work incapacity and sickness absence [14], and depression estimated to be the leading cause of disability at work by 2020 [15], a recent systematic meta-review found that there no workplace mental health interventions that could be considered evidence-based [16].

The past decade has seen an explosion in the delivery of eHealth interventions targeting common mental illnesses such as depression and anxiety, as well as those focused upon broader concepts of stress and distress [17] through Apps and via the Internet. The components of these interventions have included Cognitive Behavioural Therapy, Stress Management, Mindfulness approaches and Cognitive training. Some eHealth interventions offer fully unguided self-help programs while others involve supported guidance throughout and are thus more costly [18]. Their effectiveness has been examined in both general population and in clinical settings. Two previous systematic review and meta-analysis found that eHealth interventions delivered to general population could both reduce and prevent depression and anxiety [11,19]. Similar results were found in a systematic review and meta-analysis of eHealth interventions in a clinical setting [20].

Thus there is currently ample evidence for the short and long term benefits for eHealth delivered Cognitive Behavioural Therapy (CBT) for treating anxiety and depressive conditions in both general population and clinical settings [21–23], with greater improvement shown for those interventions that include guidance throughout the intervention period [21,24]. Evidence has also emerged for the effectiveness of Mindfulness based eHealth interventions in improving symptoms in both unselected and symptomatic people [25].

However, we know employed people differ systemically from both general or clinical populations used in most eHealth studies (in terms of symptom profile, risks, function and response [26]) Therefore, this evidence may not be generalisable to the occupational setting.

Many workplaces now provide employees with access to mental health assistance, usually through Employee Assistance Programs (EAPs), providing employees with a range of integrated services including short term counseling, management consultations, organisational and team interventions, often with aggregate reporting to the organisation [27]. EAPs are estimated to cost US workplaces up to US$40.00 per person [28]. To mitigate the cost of these, many organisations are now providing eHealth interventions and applications into their workplace health management systems for employees, either by creating their own programs for internal use or buying commercially available products [29,30]. However there is no guidance on whether eHealth interventions are effective in the workplace, or even whether they may cause harm [30]. Given this gap in the evidence we undertook a systematic review of the effectiveness of eHealth interventions delivered to employees through the workplace with the aim of informing organisations and employees that eHealth interventions targeting mental ill-health and work stress are safe and effective.

## Methods

This work complies with the Preferred Reporting Items for Systematic Reviews and Meta-Analyses (PRISMA) guidelines [31].

We aimed to identify all published, peer reviewed, clinical trials including randomized controlled trials (RCTs), controlled trials & pre/post trials using an eHealth intervention targeted at employees that reported outcomes on a standardized mental health measure of depression, anxiety and/or stress.

## Eligibility criteria

### Inclusion

We included only studies evaluating the outcome of an eHealth based intervention, defined as a therapeutic intervention delivered through a website, smartphone or tablet App, focused on the mental health of employees. Participants of the studies had to be in current paid employment and working age adults between 18 to 65 years of age. Included studies were peer-reviewed articles published in English.

### Exclusion

Studies were excluded from the review if they focused on volunteer workers, unemployed participants, general or clinical population studies that did not mention being targeted to employees or in a workplace setting, examined non-mental health outcomes, or used telephone and/or email interventions only. Studies without a control group were also excluded from the meta-analysis.

### Search strategy

A thorough literature search was conducted using the electronic databases MEDLINE, PsycINFO, Cochrane Register of Controlled Trials (CENTRAL) and EMBASE for relevant articles published from 1975 (the first eHealth intervention for employees was identified in 2004 [32]) until 17 November 2016. Keywords were searched relating to ‘workplace’ and ‘intervention’ and ‘outcome’ and ‘study design’ and an example of a search strategy for these databases is displayed in S1 Table. To increase coverage for relevant databases, we also manually screened the table of contents of the Journal of Medical Internet Research, Journal of Internet Interventions, Occupational and Environmental medicine and the Journal of Occupational Environmental Medicine, as well as the reference list of included studies.

### Study selection criteria

#### Identification

After duplicates were removed, authors (ES) screened all titles and abstracts to identify potentially relevant studies. Abstracts and Full-text versions of potentially eligible studies were independently assessed by two investigators (ES and IC). Disagreements were adjudicated in conjunction with the senior author (NG).

#### Data collection and coding of outcomes

We collected the mean, and standard deviation (SD) of standardized measures of (dis)stress, depression and/or anxiety, and sample size (n) of participants in each arm (intervention and control) at baseline and each follow-up. Where studies used a number of measures, these were pooled as a single composite Mental Health outcome. When SDs were not reported, we contacted the authors. Three authors were contacted, and two of those responded with relevant data [33,34]. In one study [35] the SD could not be obtained from the paper or the authors; effect sizes for this study were derived from 2-tailed p-value for Group × Time interaction and sample sizes. One author did not respond and we were unable to obtain data so this study was excluded from the meta-analysis [36].

Where studies reported results of the same intervention in different papers (e.g., a post intervention and follow-up study), papers were grouped together and treated as one study and we used the first follow-up outcome point in the analysis.

We then categorized the studies by intervention type, in which three main types were identified from the primary paper as to the main component of the intervention: Cognitive Behavioural Therapy (CBT), Stress Management, and Mindfulness-based treatments, and by whether the intervention was given to unselected employees (universal) or targeted to those selected for higher symptom scores or another indication of poorer mental health (indicated). Sensitivity analysis results were also assessed by whether the intervention was unguided (self-help) or guided (feedback, rather than just technical support, as this is known to affect the outcome [37].

#### Quality assessment and risk of bias within studies

The quality of the identified studies was assessed using the Downs and Black checklist [38]. Specifically developed for public health, and used previously in similar reviews in the field [16], the Downs and Black checklist demonstrates good inter-rater reliability (r = 0.75) and strong criterion validity (r = 0.90) [11]. The checklist has 27-items to score within five sub categories: reporting, external validity, internal validity -assessing risk of bias & confounding, and power. The checklist was modified to simplify the power item to a score of either zero = no or one = yes based on whether the studies reported sufficient power to detect a clinically significant effect, as reported in previous studies [11,39]. The maximum score for the modified checklist was 28 with most items reporting on yes = one, no = zero or unable to determine = zero, with the exception of one question that used yes = two, partially = one, and no = zero. The first author (ES) individually scored each included paper. Scores were pooled into four categories as used in other reviews [11]: Excellent = 26 to 28, Good = 20 to 25, Fair = 15 to 19 and Poor = 14 and less. Any rated poor were reviewed by an independent research assistant and discrepancies adjudicated by the senior author (NG) and excluded.

As a further standardized measure we assessed Risk of Bias using the Cochrane Guidelines [40]. For this analysis five of the six items (sequence generation, allocation concealment, assessor blinding, incomplete outcome data and selective outcome reporting) were used to assess bias as all of the trials included in the Meta-analysis had only a wait list control group, and it would be impossible for participants and personnel to be blinded to the arm that they were randomized to (S1 Fig).

### Statistical analysis

The summary measure was the standardized mean difference (SMD, calculated as Hedge’s g), of change in the effect sizes between baseline and each follow-up. A positive effect size (SMD) indicated that the intervention group had superior effects to the control group. Using standard measures of effect size, SMDs of 0.2 were considered small, 0.5 moderate, and 0.8 large [41]. Precision of effect sizes was estimated using 95% confidence interval (CI). Pooling of effect sizes across studies was done using random effects model in Comprehensive Meta-Analysis V3. Random effects analysis was used as we could not make the assumption that these studies represented a common effect as the different types of interventions delivered may result in large heterogeneity [42].

Analyses were conducted in three stages, first, we combined results of all studies to achieve a combined effect size at each time point, immediately post-intervention and at follow-up. We then estimated pooled effect sizes for each of the three intervention types (CBT, Stress Management, and Mindfulness based interventions). Studies targeting unselected employees were analysed separately from those targeting ‘symptomatic’ (selected) employees such as those having reported increased ‘stress’ or having a mental health condition at baseline measures indicated for intervention. Studies were then stratified using the post-intervention and follow-up combined SMD for each study in each sub-group.

Heterogeneity of real effects across studies was quantified using the I^2^ statistic to estimate the proportion observed variance not due to sampling error.

To assess small study effect (in the case where at least 10 studies were available for analysis) we used a funnel plot for the overall effects and each of the grouped intervention types which compared the outcome effects with their standard errors. We used Egger regression test to further examine asymmetry of the funnel plot [43] with a statistical significance based on a *P*-value less than 0.05. In the case where at least 10 studies were available for analysis and a small study effect was found we used a Duval and Tweedie trim and fill to quantify the magnitude of small study effect [44].

## Results

### Search results

**Fig 1:**
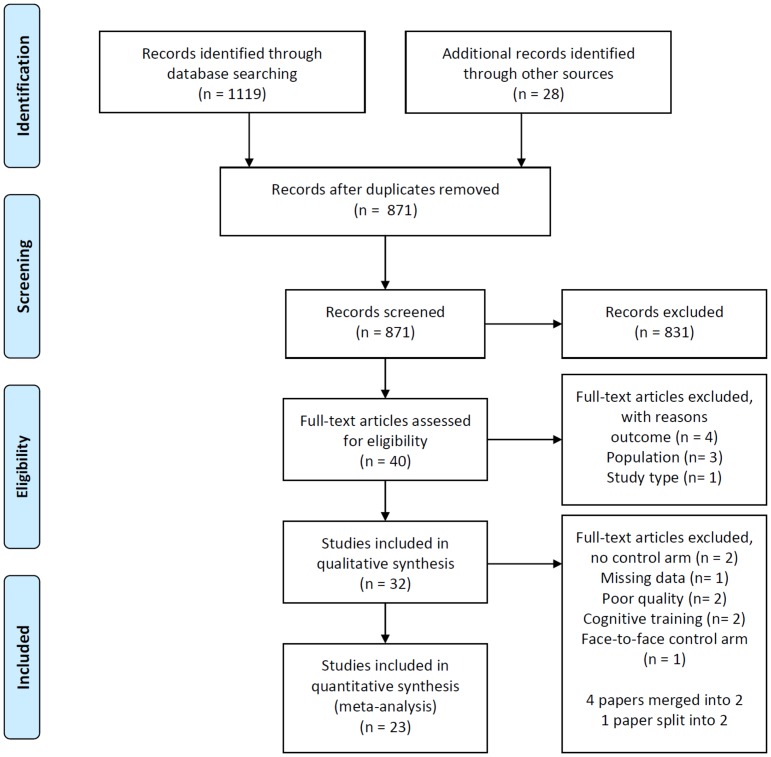
Flow chart of studies selected.

The search strategy identified a total of 1147 titles (Fig 1). After the removal of duplicates (n = 276), 871 titles and abstracts were reviewed by author (ES). Of those, 723 studies were excluded on the basis of the inclusion/exclusion criteria, leaving 108 studies potentially relevant to the research question. The 108 abstracts were examined by the two independent researchers (ES and IC), and 68 articles were excluded. Both researchers independently examined the full text of 40 articles, where a further 8 articles were excluded. The 32 remaining articles were identified as meeting criteria for quality assessment [32–37,45–71]. Four papers were identified as reporting on different follow-up periods and were merged [48,49] and [50,53]. After the quality assessment of all studies using the Down’ & Black Checklist, two studies were excluded from the analysis as they were found to be of ‘poor’ quality [45,55]. Two papers did not have a control group [56,57], and one study used a face-to-face control group [68]. One study [58] included two intervention groups, which were treated as two studies in the meta-analysis. The two intervention groups’ SMDs were computed and the mean (n) of the control group was evenly divided among each intervention group to ensure that control participants were only included once in the analysis. The two studies using cognitive training [59,60] were not included for the meta-analysis as the outcomes were not standardized mental health outcomes. One paper was excluded due to missing data [36]. This approach left 23 studies to be analysed in the meta-analysis, 22 of which were randomized controlled trials and a “controlled” trial [33].

Table 1 shows the details of each included intervention. Eleven studies were identified to have a CBT intervention [32,33,35,45–53,56,61], six studies described themselves as Stress Management [34,36,62–66], and six studies used Mindfulness-based approaches [57,58,67,69–71]. Of the CBT interventions, nine were web-based interventions while two were smartphone Apps. All of the Stress Management interventions were web-based interventions. Of the Mindfulness based interventions, five were web-based and one was on a smartphone App (Table 1).

**Table 1:** Studies included sorted firstly by intervention type and secondly by publication year.

Study (ref #)	Type of intervention	Included in Meta-analysis	Targeted to symptomatic or high stressed participants?	Participants	Intervention, Duration + (n)	Control, n	Outcome(s) + Measure(s)	Baseline score (m,SD,n)	Post-intervention (from baseline)	Results—Post-intervention (m,SD,n)	Follow-up (from baseline)	Results—Follow-up (m,SD,n)
**CBT based intervention**												
Grime. (2004) (32)*	CBT	Yes	Yes	London, Occupational Health department. employees with >10 days sick leave in the past 6 months due to mental illness (n = 48)	‘Beating the Blues’ a computerised CBT program for Depression and anxiety, 8 online sessions which last approximately an hour per week (n = 24)	WLC (n = 24)	Primary: Anxiety & Depression (HADS)	Intervention: Depression (7.96,3.43,24) Anxiety (11.75,3.87,24) Control: Depression (10.63,4.13,24) Anxiety (14.04,4.34,24)	2 months	Depression Intervention (5.38, 3.93, 16) Control (8.61,3.86,23) Anxiety Intervention (10.13, 4.65,14) Control (12.00,4.31,23)	3 months	Depression Intervention (5.00,4.12,13) Control (7.32,5.08,19) Anxiety Intervention (8.69,3.50,13) Control (9.47,5.26,19)
											6 months	Depression Intervention (5.07,4.57,14) Control (6.21,4.22,19) Anxiety Intervention (8.86,4.35,14) Control (9.16,4.37,19)
Shimazu. et al. (2005) (45)*	CBT	Yes	No	Japan, any employee company wide in a construction machinery company (n = 225)	One-month web-based psycho-education based on social cognitive theory. Self-based program, 3 phases (5 chapters), (n = 112)	WLC (n = 113)	Primary: Stress (BJSQ)	Intervention: Stress (36.80,9.88,112) Control: Stress (38.30,9.39,113)	5 weeks	Stress Intervention (35.70,5.68,105) Control (37.60,6.76,107)	nil	not measured
Hasson. et al. (2005) (35)* ^	CBT	Yes	No	Sweden, IT and media companies general employees (n = 317)	12 month open access. Web-based self-help exercises—developed specifically for this study. Uses CBT techniques (n = 129)	WLC + info (n = 174)	Primary: Stress: (VAS)	Not reported	12 months	Not reported	nil	not measured
Billings. et al (2008) #	CBT	Yes	No	USA, employees from a technology company on a health and activity promotion program (n = 309)	3 month web-based. Participants could review sections more than once. Uses CBT—website is tailored around baseline answers for each participant depending on risks and needs (n = 154).	WLC (n = 155)	Primary: Stress: (symptoms of distress scale) Depression: (CES-D) Anxiety: (BAI)	Intervention: Stress (17.52,4.53,154) Depression (33.77,13.46,115) Anxiety (28.87,7.82,154) Control: Stress (16.81,3.78,155) Depression (32.15,10.08,155) Anxiety (27.98,7.43,155)	3 months	Stress Intervention (16.03,4.18,113) Control (16.50,4.35,132) Depression Intervention (31.60,13.33,113) Control (31.57,10.56,132) Anxiety Intervention (27.54,7.53,113) Control (27.30,6.19,132)	nil	not measured
Lappalainen. et al. (2013) (46) * ^	CBT	Yes	Yes	Finland, males aged 28-58yrs with exhaustion, stress symptoms, or sleeping problems (n = 23)	‘P4Well′, 3 month novel CBT intervention Delivered via multiple channels, including 3 group meetings, Internet/Web portal, mobile phone applications, and personal monitoring devices. (n = 11)	WLC (n = 12)	Primary: Depression–(BDI 21-item)	Intervention: Depression (14.64,7.61,11) Control: Depression (13.33,9.24,12)	3 months	Depression Intervention (6.20,3.30,11) Control (9.30,7.10,12)	6 months	Depression Intervention (6.20,3.30,11) Control not measured
Boiler. et al. (2014) (47)* ^	CBT	Yes	No	Netherland, Nurses and allied health professionals general employees (n = 423)	Based on screening results participants were offered a tailored web-based interventions ranging from 4 to 8 weeks (n = 212)	WLC (n = 211)	Primary: Depression (BSI), Secondary: Anxiety (BSI)	Intervention: Depression (0.31,0.32,212) Anxiety (0.21,0.24,212) Control: Depression (0.30,0.35,212) Anxiety (0.25,0.32,211)	3 months	Depression Intervention (0.24,0.32,143) Control (0.26,0.35,82) Anxiety Intervention (0.16,0.24,143) Control (0.19,0.30,82)	6 months	Depression Intervention (0.22,0.31,138) Control (0.29,0.35,70) Anxiety Intervention (0.17,0.24,138) Control (0.22,0.32,70)
Geraedts. et al. (2014) (two papers 48 & 49)* ^	CBT	Yes	Yes	Netherland, employees from banking companies with elevated Depression (n = 231)	Web-based guided self-help intervention, called Happy@Work, 6 weekly sessions consisted of problem solving techniques and CBT, complete an assessment each week to move on (n = 116)	WLC (n = 115)	Primary: Depression (CES-D), Secondary: anxiety (HADS)	Intervention: Depression (25.70,7.50,116) Anxiety (10.60,3.80,116) Control: Depression (26.10,7.00,116) Anxiety (10.20,3.20,116)	2 months	Depression Intervention (15.80,10.60,116) Control (18.30,9.10,115) Anxiety Intervention (7.60,3.80,116) Control (8.30,3.60,115)	12 months	Depression Intervention (15.70,11.30,116) Control (16.20,10.70,115) Anxiety Intervention (6.80,4.10,116) Control (6.80,4.00,115)
Imamura. et al. (2014) & (2015) (two papers 50 & 53) * ^	CBT	Yes	Yes	Japan, IT employees with no Major Depressive Disorder in last month or treated for mental health problems in last month (n = 762)	6 week internet-based computerized CBT. 6 lessons, one lesson per week, approx. 30 mins each. Each lesson had homework. Given 10 weeks to complete study in whole (n = 381).	WLC + info (n = 381)	Primary: Depression (BDI-21), Secondary: stress (K6), anxiety (DASS)	Intervention: Stress (5.60,4.60,381) Depression (11.90,8.00,381) Anxiety (88.00,21.50,381) Control: Stress (5.60,4.70,381) Depression (11.80,8.00,381) Anxiety (87.40,20.90,381)	3 months	Stress Intervention (5.60,4.60,270) Control (5.80,4.70,336) Depression Intervention (10.70,8.60,270) Control (11.70,8.30,336) Anxiety Intervention (87.40,21.70,270) Control (88.50,20.90,336)	6 months	Stress Intervention (5.70,4.80,272) Control (6.40,5.10,320) Depression Intervention (11.30,9.60,272) Control (12.10,8.70,320) Anxiety Intervention (84.90,23.30,272) Control (87.00,22.80,320)
											12 months	Stress Intervention (5.40,4.70,239) Control (5.80,4.70,272) Depression Intervention (11.20,9.40,239) Control (11.60,9.00,272)
Mori. et al. (2014) (51) * ^	CBT	Yes	No	Japan, IT employees, system engineers with high computer literacy (n = 168)	4-week intervention with homework. A Web-Based Training Program using CBT. 150 minute group class—participants use web based entries to log daily stresses (n = 85)	WLC (n = 83)	Primary: Psychological distress (K6).	Intervention: Stress (4.70,4.50,85) Control: Stress (4.80,4.50,83)	1 month	Stress Intervention (4.20,4.90,85) Control (5.00,4.80,83)	6 months	Stress Intervention (4.60,5.10,85) Control (5.63,5.00,83)
Phillips. et al. (2014) (52) *	CBT	Yes	Yes	UK, transport, health and communications sectors who reported issues with difficulty in some tasks at work (n = 637)	5 week web-based intervention ‘MoodGYM’ five one hour long modules containing CBT skills for preventing and coping with Depression (n = 318)	WLC + info (n = 319)	Primary: Depression (PHQ-9), and (Generalized Anxiety Disorder GAD)	Intervention: Stress (19.90,8.00,318) Depression (14.50,5.40,318) Anxiety (13,50.4.00,318) Control: Stress (20,70.7.00,319) Depression (14.60,5.60,319) Anxiety (13.20,5.00,319)	6 weeks	Stress Intervention (16.00,9.10,171) Control (16.50,8.60,188) Depression Intervention (9.90,6.10,164) Control (10.20,6.00,176) Anxiety Intervention (9.50,6.00,166) Control (10.20,5.70,181)	3 months	Stress Intervention (15.00,10.10,102) Control (15.90,8.60,129) Depression Intervention (9.30,6.90,97) Control (10.30,6.90,122) Anxiety Intervention (8.40,6.40,98) Control (10.10,6.50,123)
Takechi. et al. (2015) (56) $	CBT	No, no control group	No	Japan, Manufacturing company, general employees (n = 81)	10-week single group study, CBT exercise program workbook focusing on dysfunctional thoughts and problem solving, followed by 10 weeks of at home exercises.	nil	Primary: Psychological distress (K6).	Intervention: Stress (4.39,3.70,81)	10 weeks	Stress Intervention (3.53,2.70,44)	Nil	not measured
Birney. et al. (2016) (61)*	CBT	Yes	Yes	USA, Employees with mild to moderate depressive symptoms (n = 300)	6 weeks CBT mobile phone app “MoodHacker” (n = 150)	WLC (n = 150)	Primary: Depression (PHQ-9)	Intervention: Depression (13.20,4.30,150) Control: Depression (13.60,3.80,150)	10 weeks	Depression Intervention (8.80,5.10,130) Control (9.50,5.00,141)	Nil	not measured
Stress Management Interventions												
Cook. et al. (2007) (62)*	Stress Mx	Yes	No	USA, human resource employees (n = 419)	Web-based multimedia health promotion program ‘Health Connection’ for the workplace offering guidance on stress management (n = 209)	WLC + info (n = 210)	Primary: Stress	Intervention: Stress (14.21,4.90,209) Control: Stress (15.05,4.60,210)	3 months	Stress Intervention (13.70,4.90,209) Control (14.30,4.50,210)	nil	not measured
Eisen. et al. (2008) (36)*	Stress Mx	No, face-to-face control group	No	manufacturing company general employees (n = 257)	2 week online intervention: two sessions online stress management program (n = 123)	completing identical intervention instructor led workshop (n = 134)	Stress: (Occupational Stress inventory, OSI-R)	Intervention: Stress (45.80,14.90,123) Control: Stress (42.90,20.50,134)	2 weeks	Stress Intervention (38.30,9.30,24) Control (21.70,15.20,75)	1 month	Stress Intervention (33.30,16.30,15) Control (21.10,14.50,48)
Cook. et al. (2015) (34)*	Stress Mx	Yes	No	USA, Employees aged 50–68 years global IT company (n = 278)	‘HealthyPast50’ is a web-based. Open access for 3 months, 5 modules on stress and mood management, healthy eating, active lifestyle, and smoking (n = 138)	WLC (n = 140)	Primary: Stress (The Ultimate Stress Management, Self-Assessment, and Coping Guide)	Intervention: Stress (3.24,0.50,138) Control: Stress (3.20,0.40,140)	3 months	Stress Intervention (3.30,0.50,109) Control (3.34,0.50,130)	nil	not measured
Ebert. et al. (2014) (63)* ^	Stress Mx	Yes	Yes	German, teachers with elevated depressive symptoms currently employed (n = 150)	6 weeks, 5 lessons, based on problem solving techniques, internet-based problem-solving training (iPST), one lesson per week and practice problem solving skills between each lesson (n = 75)	WLC (n = 75)	Primary: depressive symptoms (CES-D), Secondary: stress (PSQ)	Intervention: Stress (0.66,0.15,75) Depression (22.76,9.20,75) Control: Stress (0.67,0.14,75) Depression (22.80,9.15,75)	7 weeks	Stress Intervention (0.55,0.20,75) Control (0.62,0.17,75) Depression Intervention (22.81,9.15,75) Control (21.20,8.37,75)	3 months	Stress Intervention (0.48,0.27,75) Control (0.56,0.25,75) Depression Intervention (15.37,8.40,75) Control (19.87,14.00,75)
											6 months	Stress Intervention (0.53,0.20,75) Control (0.60,0.16,75) Depression Intervention (14.53,13.80,75) Control (19.91,10.42,75)
Umanodan. et al. (2014) (64)*	Stress Mx	Yes	No	Japan, general employees in a manufacturing company (n = 266)	6 week Computer based stress management training (SMT) 6 lessons (one per week)—self paced. 2-phased learning process (n = 142)	WLC (n = 121)	Primary: Stress (BJSQ)	Intervention: Stress (2.00,0.50,142) Control: Stress (2.10,0.56,121)	9 weeks	Stress Intervention (1.90,0.45,142) Control (2.00,0.55,121)	19 weeks	Stress Intervention (2.00,0.04,142) Control (2.00,0.55,121)
Stansfeld, S., et al. (2015) (65)* ^	Stress Mx	Yes	No	UK, manager and general employees of the NHS Mental Health Trust (n = 275)	Online health promotion program based around understanding stress through a series of linked topics with case examples. Six fortnightly modules for 3 months (n = 216)	WLC (n = 59)	psychological distress (GHQ-12)	Intervention: Stress (2.80,3.50,216) Control: Stress (3.20,3.40,59)	3 months	Stress Intervention (2.90,3.50,216) Control (2.90,3.40,59)	nil	not measured
Heber. et al. (2016) (66)* ^	Stress Mx	Yes	Yes	Germany, mainly recruited by a large health insurance company of highly stressed employees (n = 264)	7 sessions. 1–2 sessions per week. web-based Internet stress management interventions (iSMI) problem solving, emotion regulation strategies + booster session (n = 132)	WLC (n = 132)	Primary: Stress (PSS). Secondary: Depression (CES-D, HADS)	Intervention: Stress (25.90,3.90,132) Depression (23.34,8.50,132) Anxiety (11.20,3.30,132) Control: Stress (25.15,3.96,132) Depression (23.77,7.60,132) Anxiety (10.70,3.40,132)	7 weeks	Stress Intervention (17.90,6.17,132) Control (22.90,6.10,132) Depression Intervention (15.60,9.10,132) Control (21.40,8.80,132) Anxiety Intervention (7.80,3.90,132) Control (10.30,3.50,132)	6 months	Stress Intervention (16.08,6.00,132) Control (22.10,5.80,132) Depression Intervention (13.80,7.70,132) Control (31.50,8.50,132) Anxiety Intervention (6.73,3.40,132) Control (9.65,3.60,132)
Mindfulness Based Interventions												
Glück. et al. (2011) (67)*	Mindfulness based	Yes	No	Austria, Germany & Switzerland General employees of universities, car dealership, broadcasting station, and health care consulting companies in (n = 50)	web based mindfulness training 13 days two modules each module lasted for 6 days with 20 mins per day (n = 28)	WLC (n = 21)	Primary: Stress (PSQ)	Intervention: Stress (40.06,16.40,28) Control: Stress (35.10,13.40,21)	2 weeks	Stress Intervention (34.40,15.00,26) Control (34.72,15.35,18)	3 months	Stress Intervention (27.90,11.20,19) Control group not tested
Wolever. et al. (2012) (68)*	Mindfulness based	No, face-to-face control group	Yes	USA, insurance carrier employees with elevated stress levels (n = 96)	12 week online interventions lasted 1hr per week (n = 52)	In person mindfulness training (n = 44)	Primary: Stress (PSS), Secondary: Depression (CES-D)	Intervention: Stress (24.50,0.50,52) Depression (19.60,1.30,52) Control: Stress (24.85,0.53,44) Depression (20.00,1.40,44)	3 months	Stress Intervention (14.90,0.80,52) Control (16.94,0.86,44) Depression Intervention (11.10,1.20,52) Control (14.23,1.30,44)	nil	not measured
Ahtinen. et al. (2013) (57)$	Mindfulness based	No, no control group	No	Finland, general employees from a technology University (n = 15)	4 week mobile phone app intervention “Oiva” mental wellness training app. With 4 intervention modules to be taken weekly	nil	Primary: Stress (PSS)	Intervention: Stress (3.10,0.20,15)	1 month	Stress Intervention (2.50,0.10,15)	nil	not measured
Aikens. et al. (2014) (69)*	Mindfulness based	Yes	No	USA, general employees (n = 90)	7 week web-based online mindfulness training program. 1 hour per week modules (n = 44)	WLC (n = 45)	Primary: Stress (PSS-14)	Intervention: Stress (24.50,6.30,44) Control: Stress (24.80,8.16,45)	7 weeks	Stress Intervention (18.00,7.00,36) Control (23.30,8.50,42)	6 months	Stress Intervention (18.80,6.70,31) Control (19.81,7.40,32)
Ly. et al. (2014) (70)*	Mindfulness based	Yes	No	Sweden, middle managers or have staff responsibilities in the private sector (n = 73)	6 week smartphone app mindfulness based. 6 modules one for each week. each module had an audio lecture, text & exercises (n = 36)	WLC (n = 37)	Primary: Stress (PSS-14).	Intervention: Stress (24.30,8.30,36) Control: Stress (24.50,5.90,37)	6 weeks	Stress Intervention (19.50,7.30,36) Control (23.30,8.00,37)	nil	not measured
Mak. et al. (2015) (58)*	Mindfulness based	Yes	No	China, University staff—general employees (n = 321)	two arm intervention: 8 week online mindfulness training, one lesson per week that took 23–30 (107)	WLC (n = 107)	Primary: Stress (PSS-10), Depression, Anxiety (DASS-21)	Intervention: Stress (1.70,0.60,107) Depression (6.10,6.30,107) Anxiety (6.70,5.20,107) Control: Stress (1.63,0.70,55) Depression (5.47,7.60,55) Anxiety (6.20,8.00,55)	2 months	Stress Intervention (1.65,0.50,58) Control (1.62,0.70,48) Depression Intervention (5.70,6.80,58) Control (5.60,7.70,48) Anxiety Intervention (6.60,5.40,58) Control (5.70,7.30,48)	3 months	Stress Intervention (1.70,0.50,44) Control (1.63,0.70,24) Depression Intervention (6.80,7.20,44) Control (5.90,8.12,24) Anxiety Intervention (7.30,6.40,44) Control (5.70,7.60,24)
					the second group had the identical training plus HAPA (health action process approach) to test enhanced efficacy (n = 107)			Intervention: Stress (1.80,0.60,107) Depression (6.95,7.70,107) Anxiety (7.50,6.60,107) Control: Stress (1.63,0.70,54) Depression (5.47,7.60,54) Anxiety (6.20,8.00,54)		Stress Intervention (1.70,0.60,58) Control (1.62,0.70,48) Depression Intervention (6.50,7.40,58) Control (5.60,7.70,48) Anxiety Intervention (6.30,6.60,58) Control (5.70,7.30,48)		Stress Intervention (1.70,0.60,37) Control (1.63,0.70,24) Depression Intervention (6.90,7.70,37) Control (5.90,8.12,24) Anxiety Intervention (6.50,6.60,37) Control (5.70,7.60,24)
Allexandre. et al. (2016) (71)*	Mindfulness based	Yes	No	USA, General employees from a corporate call centre (n = 91)	8 week web-based educational program based on mindfulness meditation. 1 session per week (n = 54)	WLC (n = 37)	Primary: Stress (PSS-10)	Intervention: Stress (25.60,5.40,54) Control: Stress (25.40,5.70,37)	2 months	Stress Intervention (19.80,7.60,30) Control (24.00,7.20,25)	4 months	Stress Intervention (19.40,7.70,27) Control (22.50,7.20,20)
Cognitive/Assertion Training Interventions												
Abbott. et al. (2009) (59)*	Cognitive training	No, cognitive training	No	Australian Industrial organisation general employees (n = 53)	Internet-based program teaching resilience through cognitive training. 7 core modules (n = 26)	WLC (n = 27)	Primary: Depression, Anxiety, Stress: (DASS-21)	Intervention: Stress (10.70,7.40,26) Depression (5.30,5.10,26) Anxiety (2.30,2.20,26) Control: Stress (7.60,6.90,27) Depression (3.78,5.40,27) Anxiety (2.07,2.80,27)	10 weeks	Stress Intervention (9.70,6.00,26) Control (1.56,2.40,27) Depression Intervention (4.70,4.90,26) Control (3.70,5.30,27) Anxiety Intervention (2.30,2.20,26) Control (6.30,4.90,27)	nil	not measured
Borness. et al. (2013) (60)*	Cognitive training	No, cognitive training	No	Australian Public Sector general employees (n = 135)	16 week of online Cognitive Training based around, memory, attention, language, executive function, program is called ‘Spark!’ with three 20 minute sessions per week (n = 67)	active control program (n = 68) general knowledge information	Primary: Stress (JSS, Secondary: Depression, Anxiety, DASS-42)	Intervention: Stress (10.70,6.70,67) Depression (6.20,6.20,67) Anxiety (5.40,5.60,67) Control: Stress (11.00,8.80,68) Depression (7.40,6.80,68) Anxiety (6.60,5.80,68)	4 months	Stress Intervention (10.90,7.90,58) Control (5.80,8.20,62) Depression Intervention (7.30,6.90,58) Control (6.80,7.60,62) Anxiety Intervention (6.20,7.10,58) Control (6.60,11.50,62)	nil	not measured
Yamagishi. et al. (2007) (54)$	Assertion training	No, poor quality + no control group	No	Japan, shift nurses (n = 25)	web-based assertion training (70 minutes) ‘Internet navigware’: pre, post & follow-up. Training was accessible for 3 weeks (n = 25)	nil	Primary: Stress, Depression–(JSBQ)	Intervention: Stress (2.21,0.73,25) Depression (38.52,9.31,25)	3 weeks	Stress Intervention (2.11,0.72,25) Depression Intervention (36.84,8.81,25)	1 months	Stress Intervention (2.01,0.77,25) Depression Intervention (37.44,10.74,25)
Yamagishi. et al. (2008) (55)*	Assertion training	No, poor quality	No	Japanese shift working Nurses (n = 60)	9 weeks sixty minute web-based training was provided weekly (n = 30)	WLC (n = 30)	Primary: Stress, Anxiety, Depression—JSBQ)	Intervention: Stress (2.54,0.67,30) Depression (1.85,0.70,30) Anxiety (2.18,0.80,30) Control: Stress (2.35,0.80,30) Depression (1.89,0.90,30) Anxiety (2.03,0.80,30)	5 weeks	Stress Intervention (2.58,0.60,30) Control (2.79,0.80,30) Depression Intervention (1.93,0.70,30) Control (2.04,0.80,30) Anxiety Intervention (2.26,0.70,30) Control (2.33,0.80,30)	9 weeks	Stress Intervention (2.60,0.70,30) Control (2.67,0.70,30) Depression Intervention (1.71,0.50,30) Control (1.78,0.60,30) Anxiety Intervention (2.00,0.69,30) Control (2.10,0.80,30)

Key:

* Randomised controlled trial,

^#^ Controlled Trial,

^$^ Pre-intervention post-intervention,

^ Guided,

Questionnaires used: Hospital Anxiety Depression Scale (HADS), Brief Job Stress Questionnaire (BJSQ), Centre of Epidemiologic Studies Depression scale (CES-D), Beck Anxiety Inventory (BAI), Visual Analogue Scale (VAS), Beck’s Depression Inventory (BDI-21), Brief Symptom Inventory (BSI), Kessler 6 (K6), Depression Anxiety Stress Scales DASS, 21 & 42), Patient Health Questionnaire-9 (PHQ-9), Work and Social Adjustment Scale (WSAS), Perceived Stress Scale (PSS, 10 &14), Patient Satisfaction Questionnaire (PSQ), General Health Questionnaire (GHQ-12), Job satisfaction Survey (JSS), Job Stress Burnout (JSBQ)

### Meta-analysis results

#### Overall effect of employee eHealth interventions compared to control conditions

**Fig 2:**
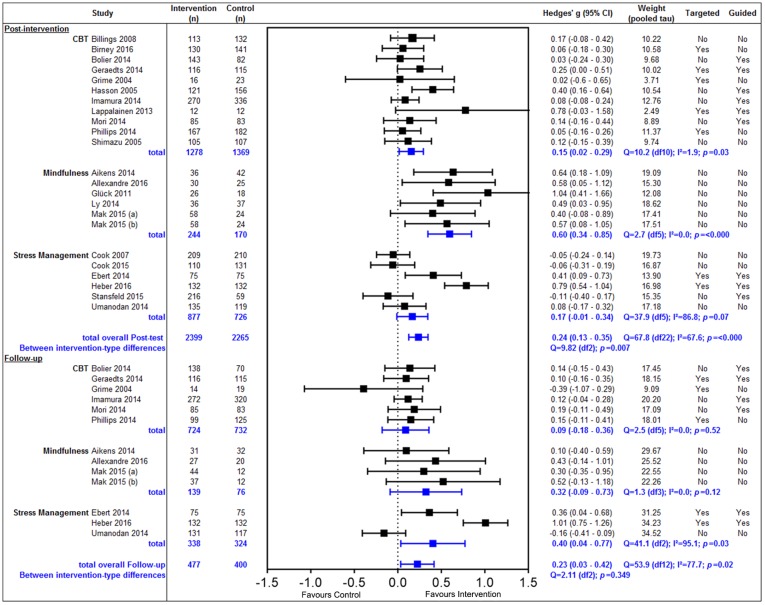
Effect of eHealth interventions in the workplace.

For the 23 trials identified (Fig 2), there was a small, significant positive overall effect post intervention (g = 0.24, 95% CI 0.13 to 0.35, p = <0.00). However, moderate to large heterogeneity was detected (I^2^ = 67.6%), and there was a significant difference between the effect of intervention types (Q = 9.82 (df2), p = <0.00).

When stratified by intervention type, CBT (n = 11) showed a very small, significant positive effect size and virtually no heterogeneity (g = 0.15, 95% CI 0.02 to 0.29, p = 0.03, I^2^ = 1.9%). Mindfulness based interventions (n = 6) showed a moderate to large positive effect size and no heterogeneity (g = 0.60, 95% CI 0.34 to 0.85, p = <0.00, I^2^ = 0.0%). Stress Management interventions (n = 6) had a non-significant small positive effect size with large heterogeneity (g = 0.17, 95% CI -0.01 to 0.34, p = 0.07, I^2^ = 86.8%).

At follow-up, there was an overall small significant positive effect (g = 0.23, 95% CI 0.03 to 0.42, p = 0.02) and again moderate to large heterogeneity was detected (I^2^ = 77.7%). When stratified by intervention type, CBT interventions (n = 6) did not maintain the post-intervention effect (g = 0.09, 95% CI -0.18 to 0.36, p = 0.52, I^2^ = 0.0%). Mindfulness intervention still showed a small positive, but not statistically significant effect (g = 0.32, 95% CI -0.09 to 0.73, p = 0.12, I^2^ = 0.0%). Stress Management interventions (n = 3) showed a moderate statistically significant positive effect, however large heterogeneity was detected (g = 0.40, 95% CI 0.04 to 0.77, p = 0.03, I^2^ = 95.1%). There was no significant difference between the intervention types detected (Q = 2.11 (df2), p = 0.35).

#### Universal vs targeted interventions

**Fig 3:**
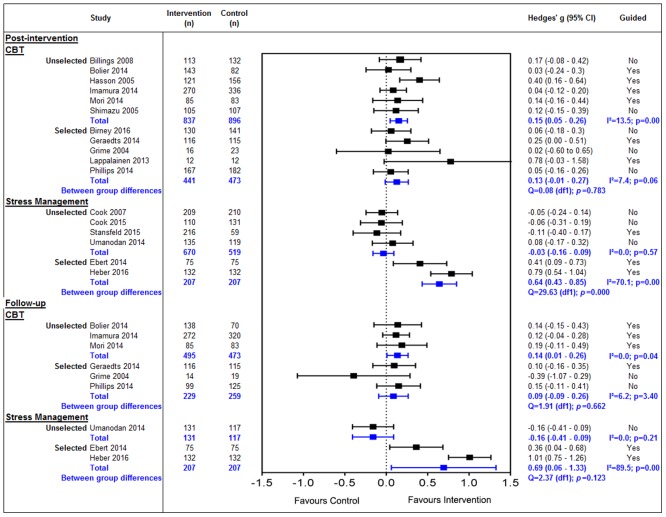
Sub-group analysis of the effect of eHealth interventions in the workplace.

There were no differences in the effect of CBT (Fig 3) at post-intervention for unselected (n = 6) (g = 0.15, 95% CI 0.05 to 0.26, p = 0.00, I^2^ = 13.5%) or selected (n = 5) (g = 0.13, 95% CI -0.01 to 0.27, p = 0.06, I^2^ = 7.4%). This continued into follow-up for unselected (n = 3) (g = 0.14, 95% CI 0.01 to 0.26, p = 0.04, I^2^ = 0.0%) and selected (n = 3) (g = 0.09, 95% CI -0.09 to 0.26, p = 3.40, I^2^ = 6.2%).

However, the effect of Stress Management interventions differed between selected and unselected employees at both post-intervention and follow-up. Interventions delivered to an unselected group (n = 4) showed a very small non-significant negative post-intervention effect (g = -0.03, 95% CI -0.16 to 0.09, p = 0.57, I^2^ = 0.0%) whereas, interventions given to a selected group showed a moderate to large significant effect reflected in the significant difference between these two groups at post-intervention (Q = 29.63 (df1), p< = 0.01) and high heterogeneity (g = 0.64, 95% CI 0.43 to 0.85, p = 0.00, I^2^ = 70.1%), we can also see that At follow-up there was only study in an unselected group showing a small non-significant negative effect (g = -0.16, 95% CI -0.41 to 0.09, p = 0.21, I^2^ = 0.0%). In contrast interventions in selected groups continued to show a significant moderate to large effect size, however large heterogeneity was detected (g = 0.69, 95% CI 0.06 to 1.33, p = 0.00, I^2^ = 89.5%). All Mindfulness-based interventions were aimed at unselected employees in the studies.

#### Small study effect

The funnel plots (S2 Fig) of the 23 studies included in the meta-analysis at post-intervention (Panel A in S2 Fig) showed significant asymmetry (Egger’s intercept  =  2.53, *p*  =  0.01). After conducting a trim and fill analysis six studies were imputed; there was an adjusted effect size, which was statistically significant (g = 0.12, 95% CI = 0.01 to 0.25) suggesting greater effect in smaller studies (Panel B in S2 Fig). There was no significant asymmetry in the 13 studies at follow-up (Egger’s intercept  =  0.32, *p*  =  0.83) (Panel C in S2 Fig). The only sub-group that had more than 10 studies was post-intervention selected CBT (n = 11) (Panel D in S2 Fig), which showed no significant asymmetry (Egger’s intercept  =  1.05, *p*  =  0.27)

#### Overview of efficacy of intervention type and moderators of efficacy across each mental health outcome

A summary of all of the outcomes is visually presented in Table 2, a matrix showing each Hedge’s g effect size for each intervention and each moderator. Overall, there is evidence for the unselected and guided CBT in reducing overall symptoms, which appears to be due to the effects on depression and stress, but not anxiety. The overall effects of unselected and unguided CBT were maintained at follow up, mainly due to the follow up effects of CBT on stress.

**Table 2:** Overview of efficacy (Hedge’s g) and moderators of each mental health outcome.

Post-Intervention			Overall	Depression	Anxiety	Stress	Follow-up			Overall	Depression	Anxiety	Stress
		Overall	0.24** (n = 23)	0.25** (n = 12)	0.21 (n = 10)	0.30** (n = 18)			Overall	0.23* (n = 13)	0.21 (n = 9)	0.21 (n = 8)	0.35** (n = 10)
All Interventions	Population	Targeted	0.31** (n = 7)	0.31** (n = 7)	0.33* (n = 4)	0.42* (n = 3)	All Interventions	Population	Targeted	0.32* (n = 5)	0.28 (n = 5)	0.32 (n = 4)	0.53* (n = 3)
		Untargeted	0.21** (n = 16)	0.18 (n = 5)	0.11 (n = 6)	0.28** (n = 15)			Untargeted	0.16 (n = 8)	0.12 (n = 4)	0.10 (n = 4)	0.25 (n = 7)
	Support	Guided	0.27** (n = 9)	0.34** (n = 6)	0.29 (n = 4)	0.29 * (n = 6)		Support	Guided	0.32 * (n = 6)	0.32* (n = 5)	0.33 (n = 4)	0.45 * (n = 4)
		Unguided	0.22 ** (n = 14)	0.14 (n = 6)	0.12 (n = 6)	0.32** (n = 12)			Unguided	0.11 (n = 7)	0.00 (n = 4)	0.03 (n = 4)	0.26 (n = 6)
	Risk of bias	High	0.20** (n = 13)	0.17 (n = 4)	0.23 (n = 3)	0.23* (n = 9)		Risk of bias	High	0.11 (n = 5)	0.13 (n = 2)	0.11 (= 2)	0.12 (n = 3)
		Low	0.29** (n = 10)	0.29** (n = 8)	0.19 (n = 7)	0.37** (n = 9)			Low	0.31* (n = 8)	0.24 (n = 7)	0.25 (n = 6)	0.45** (n = 7)
CBT	Population	Targeted	0.13 (n = 5)	0.11 (n = 5)	0.15 (n = 3)	0.05 (n = 1)	CBT	Population	Targeted	0.09 (n = 3)	0.04 (n = 3)	0.15 (n = 3)	0.09 (n = 1)
		Untargeted	0.15** (n = 6)	0.12* (n = 3)	0.06 (n = 3)	0.19** (n = 5)			Untargeted	0.14* (n = 3)	0.13 (n = 2)	0.10 (n = 2)	0.15* (n = 2)
	Support	Guided	0.18** (n = 6)	0.16* (n = 4)	0.10 (n = 3)	0.18 (n = 3)		Support	Guided	0.13* (n = 4)	0.11 (n = 3)	0.11 (n = 3)	0.15* (n = 2)
		Unguided	0.10 (n = 5)	0.07 (n = 4	0.08 (n = 3)	0.14 (n = 3)			Unguided	0.08 (n = 2)	0.07 (n = 2)	0.13 (n = 2)	0.09 (n = 1)
	Risk of bias	High	0.18** (n = 7)	0.14 (n = 4)	0.14 (n = 2)	0.24** (n = 3)		Risk of bias	High	0.14 (n = 3)	0.12 (n = 2)	0.11 (n = 2)	0.19 (n = 1)
		Low	0.10 (n = 4)	0.10 (n = 4)	0.08 (n = 4)	0.10 (n = 3)			Low	0.11 (n = 3)	0.09 (n = 5)	0.12 (n = 3)	0.13 (n = 2)
Mindfulness	Population	Targeted	-	-	-	-	Mindfulness	Population	Targeted	-	-	-	-
		Untargeted	0.59** (n = 6)	0.34* (n = 2)	0.21 (n = 3)	0.68** (n = 6)			Untargeted	0.31* (n = 4)	0.04 (n = 2)	0.11 (n = 2)	0.45** (n = 4)
	Support	Guided	-	-	-	-		Support	Guided	-	-	-	-
		Unguided	0.59** (n = 6)	0.34* (n = 2)	0.21 (n = 3)	0.68** (n = 6)			Unguided	0.31* (n = 4)	0.04 (n = 2)	0.11 (n = 2)	0.45** (n = 4)
	Risk of bias	High	0.65** (n = 3)	-	0.51 (n = 1)	0.64** (n = 3)		Risk of bias	High	0.43 (n = 1)	-	-	0.43 (n = 1)
		Low	0.54** (n = 3)	0.34 * (n = 2)	0.06 (n = 2)	0.72** (n = 3)			Low	0.26 (n = 3)	0.04 (n = 2)	0.11 (n = 2)	0.48* (n = 3)
Stress Management	Population	Targeted	0.64** (n = 2)	0.63** (n = 2)	0.83** (n = 1)	0.64** (n = 2)	Stress Management	Population	Targeted	0.70* (n = 2)	0.65* (n = 2)	1.0** (n = 1)	0.75 (n = 2)
		Untargeted	-0.03 (n = 4)	-	-	-0.04 (n = 4)			Untargeted	-0.16 (n = 1)	-	-	-0.16 (n = 1)
	Support	Guided	0.37* (n = 3)	0.63** (n = 2)	0.83** (n = 1)	0.38 (n = 3)		Support	Guided	0.70* (n = 2)	0.65* (n = 2)	1.0** (n = 1)	0.75 (n = 2)
		Unguided	-0.01 (n = 3)	-	-	-0.1 (n = 3)			Unguided	-0.16 (n = 1)	-	-	-016 (n = 1)
	Risk of bias	High	-0.03 (n = 3)	-	-	-0.03 (n = 3)		Risk of bias	High	-0.16 (n = 1)	-	-	-0.16 (n = 1)
		Low	0.38* (n = 3)	0.63** (n = 2)	0.83** (n = 1)	0.39 (n = 3)			Low	0.70* (n = 2)	0.65* (n = 2)	1.0 ** (n = 1)	0.75 (n = 2)

P = <0.05*

P = <0.001**

Mindfulness in unselected and unguided formats had a moderate to large effect size (g = 0.59) in reducing overall symptoms, a small to moderate effect in reducing depression (g = 0.34), and a large effect size (g = 0.68) in reducing stress. Unguided and unselected Mindfulness had a small to moderate effect on overall symptoms at follow up, with a large effect on stress.

Stress Management in selected formats had a large effect size (g = 0.64) and moderate effects when guided (g = 0.37) in reducing overall symptoms, a large effect in reducing depression (g = 0.63), anxiety (g = 0.83) and stress (g = 0.64). These results continued at post-intervention however the effect on Stress Management was no longer significant.

## Discussion

This study provides the first comprehensive review of the published evidence for the effectiveness of a range of work-based eHealth interventions addressing the mental health of employees.

Our results were drawn from 32 trials, testing a range of online and mobile applications utilizing Cognitive Behavioural techniques, Stress Management based approaches, and Mindfulness based interventions, 23 studies were suitable for the meta-analysis.

Overall pooled eHealth interventions showed a small significant positive effect at post intervention and follow-up for reducing overall mental health (depression, anxiety and stress) in both universal and selected employees which is similar to a recent review of eHealth interventions for general population [19], providing further evidence for potential use on eHealth interventions as a low cost, alternative to face-to-face interventions for employees [4]. However, this result showed large heterogeneity, which is examined in our sub-group analysis. There is evidence of a small study effect in the overall 23 studies at post-intervention, a possible cause for this is the clinical heterogeneity between the participants in the large and small studies, the smaller studies were targeted at the selected group (n = 1328 participants) so that a favourable outcome of the selected group may be expected compared to the larger amount of participants in the universal studies (n = 2922) [72].

As planned, sub-group analyses were performed on selected and universal interventions. Mindfulness interventions were all universal and unguided interventions, and resulted in a moderately large and statistically significant effect size at post intervention, this result has a potential to make a meaningful change when considered on a population level and is a contrary result to previous findings for interventions delivered to a universal population. Previous research indicates that large effect sizes are unlikely to be found in interventions delivered to general populations who may not be unwell [19, 73]. However, this appeared to be relatively short lived with a halved effect size at follow-up.

There was no difference between universal and targeted CBT interventions, although, previous evidence suggests that selected interventions may be more effective than universal interventions [20,74,75]. Both groups showed very small if any effect at post-intervention or follow-up, indicating that CBT interventions appeared not to have any statistical benefits either universal or targeted working populations which seems to differ from large results seen in both universal [76] and clinical [77], populations. This could be due to recent evidence that shows employed people differ systemically from the general or clinical in terms of symptom profile, function and response [26]. It may also be that the setting, with propensity for perverse incentives, or perceived coercion by employers may not be an effective therapeutic milieu.

In contrast, eHealth Stress Management interventions did appear to have benefits when used in a selected fashion e.g. amongst those reporting increased stress, showing a moderately large effect at post-intervention and a moderate effect size follow-up however, large heterogeneity was detected showing that the two selected studies had a significant difference in what can assume is the sample size as the follow-up length and intervention designs were both very similar. Importantly, when the same type of Stress Management based eHealth interventions were used in unselected (universal) employees, there was no effect compared to wait list controls. Only one study reported a follow up and suggested psychological harm, which may, if replicated, be another example of well-meaning intentions going awry, as some believe to occur with psychological debriefing [78]. It is not clear why a Stress Management intervention would not be effective when used on an unselected population, though it may relate to the dangers of providing non-help seeking populations with too much information, leading them to feel more anxious and vulnerable, as suggested by the authors of these studies[79], or could reflect intra-study effects e.g. differential drop out.

The outcome measures were then analysed separately by means of mental health condition.

We can see evidence for workplace eHealth interventions that are targeted and guided with low risk of bias reducing depression overall at post-intervention. There is no evidence to suggest that any eHealth intervention in the workplace have any effect on reducing anxiety level or symptoms at either post-intervention or follow-up. For stress levels it is evident that Stress Management interventions are most effective at post-intervention. It is evident that where a high risk of bias is found: in overall interventions, CBT and Mindfulness are all due to stress outcomes, it is unclear why, but this may be due to the different self-reporting measures used to report stress.

## Strengths and limitations

The main strengths of this review are the detailed systematic search strategy, the clearly defined inclusion and exclusion criteria and the assessment of the quality of the studies. There are a number of potential limitations, first, despite widespread use, the number of studies examining eHealth interventions in the workplace was surprisingly small with only 23 studies able to be included in the meta-analysis and for some of the subgroups there were too few to draw strong conclusions. Second, although some of the studies had clearly defined the intervention type, e.g. reporting only using CBT approaches, other interventions prioritised one approach in a more eclectic intervention, e.g. using “Mindfulness with aspects of Stress Management”, and we had to group these studies into that dominant intervention basis. However the high heterogeneity of differences of true effects when all these eHealth interventions were combined was reduced substantially when analysed by intervention type, sometimes to zero, supporting this grouping. Where a study had several outcome measures, we decided to pool them into one ‘overall mental health’ combined category, however Table 2 shows the outcome specific moderators and each level of efficacy in the intervention groups and overall. As self-reported measures were used in all studies, our conclusions are limited to self-reported reduction of symptoms rather than a clinical diagnosis. Finally, initial screening of some titles for exclusion was only completed by one author.

## Conclusion

This review demonstrated the evidence that certain types of eHealth interventions delivered to employees via their workplace can be effective at reducing mental health and stress symptoms although the evidence base is affected by a small study effect that seems to inflate effectiveness. If an eHealth intervention is to be offered to all employees, in a universal fashion, then Mindfulness approaches appear to have a stronger effect than the other types. There is little to recommend Stress Management approaches delivered to the whole workforce, and one study suggests that providing this type of intervention may even be harmful. In contrast the use of Stress Management eHealth interventions by workers who are reporting high levels of stress may have a positive effect, despite few studies. Thus although eHealth interventions are now popular and widely promoted in workplaces it appears that some caution is required in their advocacy and uptake and more evidence is needed on the effectiveness, targeting, implementation and potential risks of these new technologies.

Several considerations for future research and practice for delivering eHealth interventions to employees are evident. First, it is important to consider what the intended populations needs from the interventions to ensure the greatest potential for benefit, for example, the diverse mental health status of the employees should be considered, and where possible interventions should be offered that best fit a range of symptoms which may entail more than one application. Second, it is important to select interventions on the basis of available evidence, as some intervention types may have little or no effect on the intended outcome or may even be potentially harmful. Third, it is important for organisations that are providing interventions to their employees consider both the effectiveness of the interventions and design features to ensure that they can be applied to both universal and selected groups of people, and to be aware that not all employees would benefit from the same type of intervention. Fourth, more research is needed in the form of randomised controlled trials to understand which type of interventions would best benefit different types of employees and mental health conditions, especially Mindfulness interventions that are guided and targeting symptomatic employees, as there is no current evidence for this group.

## Supporting information

S1 Table Example search strategy. (PDF)

S1 Fig Risk of bias within studies. (TIF)

S2 Fig Funnel plots for small study effect. (TIF)

S1 File Checklist 1_PRISMA 2009 checklist. (PDF)

S2 File Raw data file. (XLSX)
