# Supplementary material for: Effectiveness of eHealth interventions for reducing mental health conditions in employees: A systematic review and meta-analysis
Source: PLoS One. 2017 Dec 21;12(12):e0189904. doi: 10.1371/journal.pone.0189904 (PMC5739441; doi:10.1371/journal.pone.0189904)
Supplement: S1 Table — (PDF) [file pone.0189904.s001.pdf]

Table 1: Example search strategy terms
MEDLINE (via OvidSP) - Medical Subject Headings [MeSH]

Workplace	Intervention	Outcomes	Study design
1.employment.ti.	13.Exp Self Care/ or exp Telemedicine/ or exp Internet/ or exp Medical Records Systems, Computerized/	28.stress.tw.	40.RCT.tw.
2.job.ti.	14.telemedicine*.tw.	29.mental health.ti.	41.randomised control trial.tw.
3.work*.ti.	15.online.tw.	30.mental illness.ti	42.random allocation.tw.
4.worker*.ti.	16.internet.tw.	31.mental disorder*.ti.	43.Randomized Controlled Trials as Topic/
5.exp employment/	17.internet intervention*.tw.	32.depress*.tw.	44.clinical trial.tw
6.work place*.tw.	18.web-based.tw.	33.anxiety*.tw.	45.controlled clinical trial.tw
7.workplace.tw.	19.therapy*.tw	34.affective symptoms.sh.	46.pre post trial.tw.
8.occupation*.tw.	20.prevent*.tw.	35.depression.sh	47. 40 or 41 or 42 or 43 or 44 or 45 or 46
9.work*.tw.	21.stress management.tw.	36.anxiety.sh.	48. 12 and 27 and 39 and 47
10.employee.tw.	22.cognitive behaviour* therapy.tw	37.mental disorder*.sh.	49. limit 48 to (English language and humans and "all adult (19 plus years)")
11.manager*.tw.	23.cognitive behavior* therapy.tw	38.mental health.sh	
12.1 or 2 or 3 or 4 or 5 or 6 or 7 or 8 or 9 or 10 or 11	24.smartphone.tw.	39. 28 or 29 or 30 or 31 or 32 or 33 or 34 or 35 or 36 or 37 or 38	
	25.Therapy, Computer-Assisted/ or Computers/ or Internet/		
	26.intervention*.tw.		
	27.13 or 14 or 15 or 16 or 17 or 18 or 19 or 20 or 21 or 22 or 23 or 24 or 25 or 26		
