# Supplementary figures and images for: Effectiveness of eHealth interventions for reducing mental health conditions in employees: A systematic review and meta-analysis
Source: PLoS One. 2017 Dec 21;12(12):e0189904. doi: 10.1371/journal.pone.0189904 (PMC5739441; doi:10.1371/journal.pone.0189904)

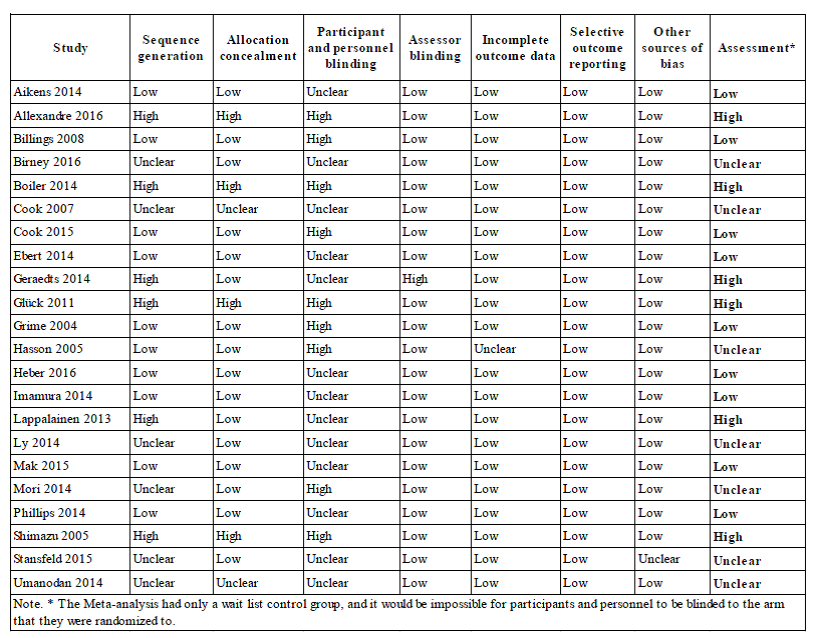

Supplement: S1 Fig — (TIF) [file pone.0189904.s002.tif]

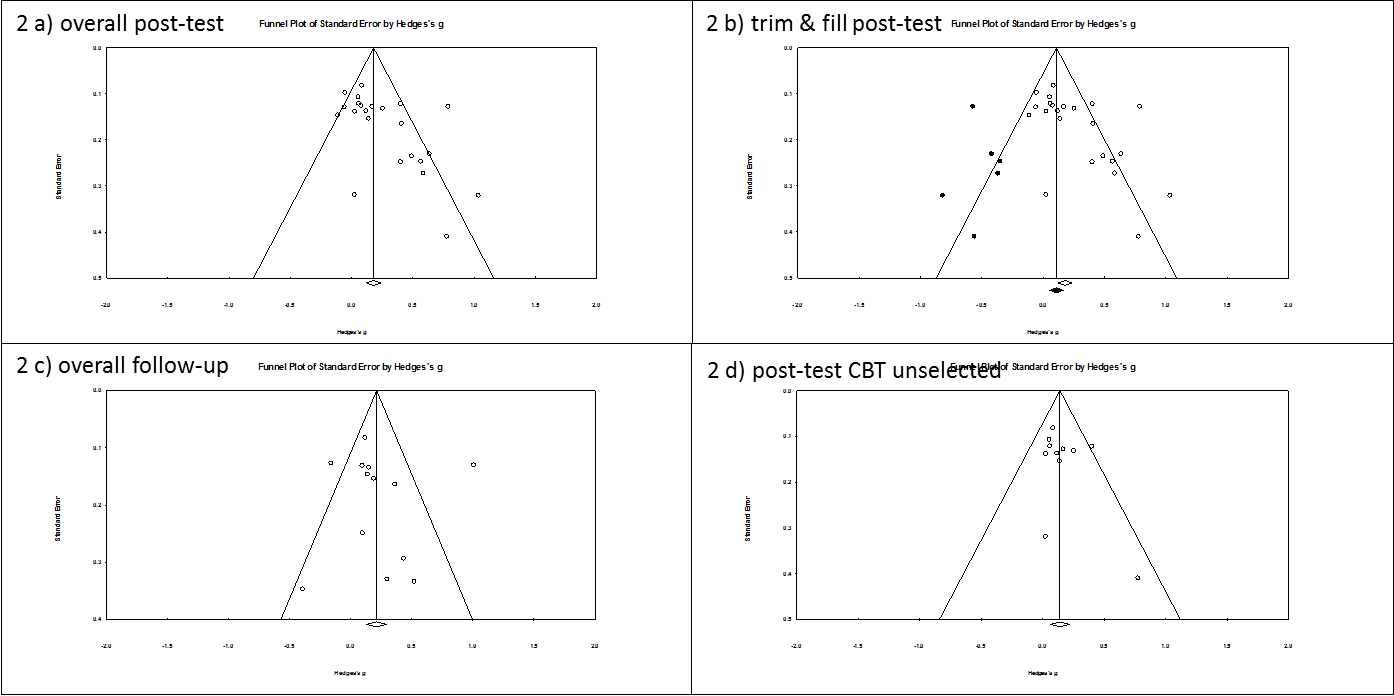

Supplement: S2 Fig — (TIF) [file pone.0189904.s003.tif]
